# Cavitation and fatal hemoptysis after immunotherapy for advanced lung adenocarcinoma: A case report

**DOI:** 10.1111/1759-7714.13578

**Published:** 2020-07-21

**Authors:** Ruijuan Wang, Kao Li, Jianjun Pi, Liwei Meng, Minli Zhu

**Affiliations:** ^1^ Department of Respiratory Medicine PLA Strategic Support Force Characteristic Medical Center Beijing China

**Keywords:** Cancer, hemoptysis, immune checkpoint inhibitors, non‐small cell lung

## Abstract

Immune checkpoint inhibitor (ICI)‐related massive hemoptysis with cavitation has rarely been identified. Here, we report a case of advanced lung adenocarcinoma with lethal bleeding after eight cycles of pembrolizumab. A 55‐year‐old male was diagnosed with stage IV non‐small cell lung cancer (NSCLC). Following confirmation of high programmed death‐ligand 1 (PD‐L1) expression of 60% cancer cells, he subsequently received pembrolizumab monotherapy. His symptoms and chest images significantly improved after four cycles of therapy. However, after eight cycles of immunotherapy, he presented with recurrence of bloody sputum and shortness of breath. Pembrolizumab was discontinued and a diagnosis of checkpoint inhibitor‐associated pneumonitis (CIP) was made. When the CIP was absorbed after glucocorticoid therapy, the patient died of sudden massive hemoptysis with cavitation in the lesion.

**Key points:**

Although checkpoint inhibitor associated pneumonitis was the leading cause of ICI‐related death, clinicians should be alerted to the finding that more attention should be given to hemoptysis attributed to ICI therapy in advanced lung cancer.

## Graphical abstract

Though checkpoint inhibitor associated pneumonitis was the leading cause of ICIs‐related death, clinicians should be alerted that hemoptysis attributed to ICIs in advanced lung cancer deserves more concern.

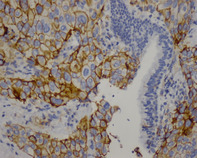

## Introduction

Compared with conventional platinum‐based chemotherapy, immune checkpoint inhibitor (ICI) therapy can significantly improve progression‐free survival (PFS) and overall survival (OS) of patients with non‐small cell lung cancer (NSCLC). Pembrolinzumab is an ICI which is recommended as first‐line treatment for advanced NSCLC showing ≥50% expression of programmed death‐ligand 1 (PD‐L1), or treatment of patients with NSCLC whose tumors express PD‐L1 ≥ 1%^.^ ^1^ As ICIs have been increasingly applied in lung cancer, immune‐related adverse events (irAEs) have aroused greater attention. Checkpoint inhibitor associated pneumonitis (CIP) is the most common serious irAE, as well as the leading cause of ICI‐related death. Here, we report a significantly rare case of massive hemoptysis with pulmonary cavitation after eight cycles of pembrolinzumab monotherapy.

## Case report

**Figure 1:**
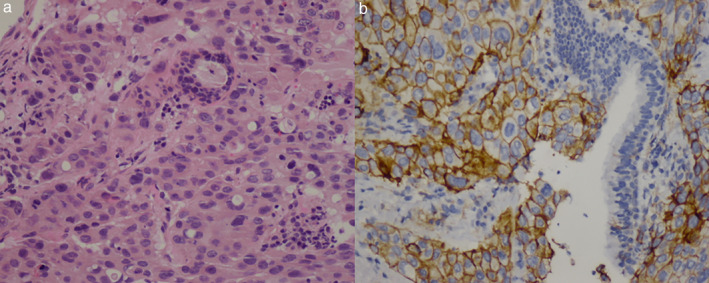
Pathological diagnosis and PD‐L1 expression. (**a**) Pathology of bronchoscopy indicated poorly‐differentiated adenocarcinoma. Hematoxylin and eosin ×200. (**b**) PD‐L1 expression in about 60% cancer cells by immunohistochemistry with SP263 clone, ×200.

**Figure 2:**
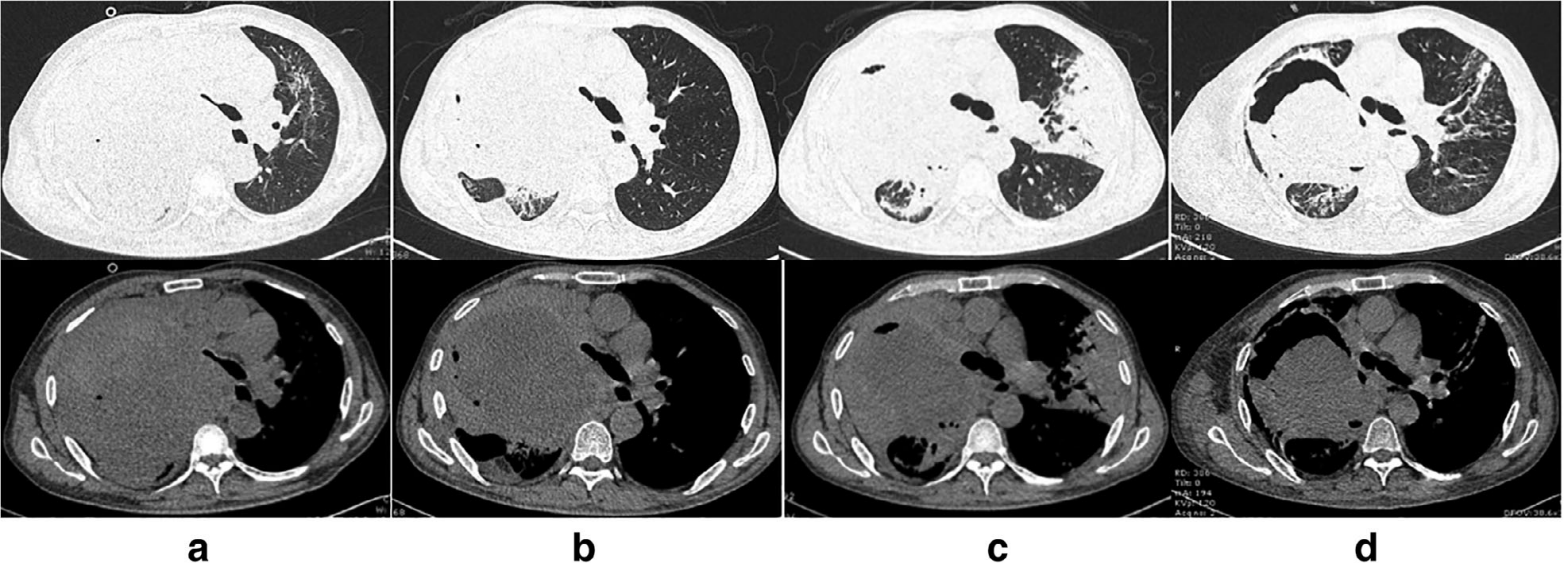
Thoracic computed tomography (CT) scan at different times. (**a**) A giant mass lesion was visible in the right lung with obvious compression of the trachea and esophagus before treatment with pembrolizumab (30 July 2019). (**b**) A shrinkage was seen in the size of the mass with a large low density area and significant compression release of trachea and esophagus after four cycles of pembrolizumab (29 October 2019). (**c**) Consolidation was seen in the left lung and small transparent area within the mass lesion in right lung after eight cycles of pembrolizumab (31 January 2020). (**d**) Cavitation in the lesion in the right lung, while consolidation in the left lung absorbed after methylprednisolone treatment (20 February 2020).

A 55‐year‐old male with a 20 pack‐year smoking status was admitted to our hospital with symptoms of dyspnea and bloody sputum in July 2019. He also complained of dysphagia and was unable to lie flat. He was diagnosed with stage IV lung adenocarcinoma (Fig 1a) in April 2019 when epidermal growth factor receptor (EGFR) and anaplastic lymphoma kinase (ALK) genomic aberrations were determined not to be present, but he was found to have a high PD‐L1 expression of 60% (Fig 1b). The patient had anaphylactic shock following brain enhanced magnetic resonance imaging (MRI) at the beginning of his illness and his previous medical history included short‐term chemotherapy without any benefit before he presented at our clinic. On admission, a significant physical examination revealed decreased breath sounds on the right side of the chest. Thoracic computed tomography (CT) scan showed a giant mass lesion in the right lung with obvious compression of trachea, right brain bronchus and part of the esophagus (Fig 2a). Given the high PD‐L1 expression of tumor cells, the patient received pembrolizumab monotherapy (100 mg/bodyweight) once every three weeks from 31 July. Subsequent surveillance with CT scan revealed shrinkage in the size of the mass but there was a large low density necrotic area in the center and significant compression release of the trachea and esophagus after four cycles (week 12) of ICI therapy (Fig 2b). All the symptoms present on admission were relieved. His efficacy was considered as a partial response (PR) which complied with the Response Evaluation Criteria in Solid Tumors (RECIST). Moreover, his performance status, abiding by the Eastern Cooperative Oncology Group (ECOG), was elevated from four prior to therapy to one. After 24 weeks of treatment with pembrolizumab, he returned to the clinic with recurrence of hemoptysis and shortness of breath, which coincided with the most serious period of the 2019 coronavirus disease (COVID‐19) in China. He had never been to Wuhan, nor had he had any contact with COVID‐19 patients. His temperature had been normal since the onset of disease, and breath sounds on the right side of his chest were significantly stronger than previously. There had been no administration of any anticoagulants or antiplatelet agent. Moreover, the results of his blood count, chemistry and coagulation tests were normal. CT scan showed progressive subpleural consolidation in the left lung after anti‐infection treatment and a small transparent area was visible within the mass lesion in the right lung (Fig 2c). Pembrolizumab was stopped later (week 25) after a diagnosis of CIP (grade 3). Methylprednisolone treatment (40 mg b.i.d.) was initiated, which was decreased with subsequent regression of the left pulmonary lesions (Fig 2d). However, the patient's symptoms were not completely relieved. Furthermore, he still presented with hemoptysis, along with cavitation in the lesion in the right lung. On week 28, the patient died of sudden massive hemoptysis.

## Discussion

Hemoptysis is one of the most frequent symptoms of lung cancer and its pathophysiology remains unclear. Pulmonary hemorrhage or hemoptysis events related to tumor treatment are often reported in clinical trials of NSCLC as the most common adverse events of antiangiogenic drugs, such as bevacizumab.^1^, ^2^, ^3^, ^4^ Some of these events are severe and even lead to fatal outcomes. However, different studies have achieved inconsistent risk factors of bleeding. In a randomized phase II study, 9% of bevacizumab‐treated patients experienced a life‐threatening bleeding event and major hemoptysis was found to be related to squamous cell histology, tumor necrosis and cavitation, and disease location close to major blood vessels.^5^ As revealed in another retrospective case control analysis suggested that tumor cavitation at baseline could be a potential risk factor in bevacizumab‐treated patients developing hemoptysis, while lesion location, size or vascular involvement appeared to be slightly related to severe hemoptysis.^6^ As revealed by a consensus from a panel of experts,^7^ patients with those risk factors of squamous cell carcinoma and a history of hemoptysis were restricted in bevacizumab use. No other clinical radiological features (including cavitation and central tumor location) could reliable predict severe pulmonary hemorrhage in bevacizumab‐treated patients, whereas major blood vessel infiltration and bronchial vessel infiltration, encasement and abutting may predict pulmonary hemorrhage.

Pneumonitis is the most common serious AE associated with pembrolizumab in lung cancer trials and the leading AE that causes pembrolizumab discontinuation.^8^ CIP is also the leading cause of ICI‐related death.^9^ Bleeding events have been rarely reported with ICIs in clinical trials and the relevant risk factors are poorly understood. Five lethal bleeding cases have been previously reported when atezolizumab was combined with chemotherapy and bevacizumab in nonsquamous NSCLC, four of which occurred in patients with potential high‐risk features, such as tumor infiltration of great vessels or cavitation.^10^ Another two fatal cases of hemoptysis have been previously reported after the first dose of pembrolizumab in patients with lung adenocarcinoma.^11^, ^12^ As suggested by the authors, the achievement of a great response and rapid tumor shrinkage after treatment with ICIs may lead to massive hemoptysis in NSCLC with central localization, vascular involvement, as well as high PD‐L1 expression.

In the present study, we report a case of lesion shrinkage after four cycles of pembrolizumab monotherapy and recurrent hemoptysis accompanied by CIP after eight cycles. The body temperature of the patient in our study was consistently normal, laboratory findings were not indicative of bacteria or fungal infection, and corticosteroids were not used before the appearance of the low density shadow and subsequent transparent area following ICI therapy. Accordingly, the cavity formation and hemoptysis could not have been associated with corticosteroid therapy, nor were they consistent with aspergillus infection. The case in our study is inconsistent with that reported by Zarogoulidis^13^ *et al*. in which a lung mass turned into an abscess together with the patient suffering from fever, infection indicated by laboratory findings and effective antibiotic treatment. Reviewing the studies of hemoptysis related to tumor treatment together with the findings in our case, we believe that the severe bleeding event was caused by the giant central location of the tumor that was significantly PD‐L1 positive, shrinkage of the mass and large area necrosis with cavity formation after immunotherapy, as well as possible vascular involvement. However, in our case, vascular involvement was unclear as it was impacted by the lack of enhanced CT as a result of MRI contrast medium allergy in the patient; in addition, patient autopsy was refused. In conclusion, this study alerts clinicians to place more emphasis on hemoptysis which could be associated with the use of ICIs in NSCLC.

## Disclosure

No authors report any conflict of interest.
